# Efficacy and Safety of a Video Game–Like Digital Therapy Intervention for Chinese Children With Attention-Deficit/Hyperactivity Disorder: Single-Arm, Open-Label Pre-Post Study

**DOI:** 10.2196/76114

**Published:** 2026-01-05

**Authors:** Mengyi Bao, Shang Feng, Jiaheng Wang, Jia Ye, Jiangping Wang, Wenyu Li, Kewen Jiang, Lin Yao

**Affiliations:** 1 Children's Hospital of Zhejiang University School of Medicine Hangzhou, Zhejiang China; 2 SDO Digital Therapeutics Innovation Center Shanghai, Shanghai China; 3 the MOE Frontiers Science Center for Brain and Brain-Machine Integration, the College of Computer Science, and the College of Biomedical Engineering and Instrument Science Zhejiang University Hangzhou, Zhejiang China; 4 Shengzhou People's Hospital (the First Affiliated Hospital of Zhejiang University Shengzhou Branch) Shengzhou, Zhejiang China; 5 Nanhu Brain-Computer Interface Institute Hangzhou, Zhejiang China; 6 Affiliated Mental Health Center and Hangzhou Seventh People's Hospital of Zhejiang University School of Medicine Hangzhou, Zhejiang China

**Keywords:** ADHD, attention, attention-deficit/hyperactivity disorder, cognitive training, digital therapy, rehabilitation

## Abstract

**Background:**

The digital therapy of attention-deficit/hyperactivity disorder (ADHD) based on a “self-adaptive multitasking training paradigm” has been developed to improve the cognitive functional impairments and attention deficits of children with ADHD. However, the efficacy and safety of such treatment for Chinese patients remain untested.

**Objective:**

This study aimed to preliminarily evaluate the actual intervention effects of a video game–like training software (ADHD-DTx) for children with ADHD aged 6-12 years as the first nationally certified digital therapeutics medical device for ADHD in China. We performed a single-arm, open-label efficacy and safety study.

**Methods:**

This is a single-arm, open-label, pre-post efficacy and safety study. A total of 97 participants were included in the analysis. Participants received digital therapy (ADHD-DTx) and basic behavioral parent training for 4 weeks (25 min/day, ≥5 times/week) without medication. The efficacy outcomes included the Test of Variables of Attention (TOVA), Swanson, Nolan, and Pelham Questionnaire, version 4 (SNAP-IV), Weiss Functional Impairment Rating Scale (WFIRS), and Conner’s Parent Symptom Questionnaire (PSQ). Safety-related events were monitored during and after the trial.

**Results:**

From day 0 (baseline) to day 28, the population TOVA Attention Performance Index exhibited statistically significant improvement (from mean –4.15, SE of the mean [SEM] 0.32 to mean –1.70, SEM 0.30; *t*_94_=–8.78; n=95; *P*<.001); the population total, inattention (AD), hyperactivity/impulsivity (HD), and oppositional defiant disorder (ODD) scores of SNAP-IV all significantly improved (total: from mean 1.33, SEM 0.05 to mean 1.09, SEM 0.05; *t*_96_=5.32; *P*<.001; AD: from mean 1.71, SEM 0.06 to mean 1.44, SEM 0.06; *t*_96_=4.44; *P*<.001; HD: from mean 1.38, SEM 0.07 to mean 1.05, SEM 0.06; *t*_96_=5.96; *P*<.001; ODD: mean 0.84, SEM 0.05 to mean 0.75, SEM 0.05; *Z*=2.47; *P*=.03; n=97); for WFIRS results, domains of “family” and “social activities” showed significant population improvement (family: from mean 0.75, SEM 0.05 to mean 0.65, SEM 0.04; *Z*=2.80; *P*=.01; social activities: from mean 0.56, SEM 0.05 to mean 0.45, SEM 0.05; *Z*=2.91; *P*=.01; n=97); for PSQ results, domains of “learning problem,” “psychosomatic problem,” “impulsivity-hyperactivity,” and “hyperactivity index” showed significant improvement (learning problem: from mean 1.72, SEM 0.06 to mean 1.57, SEM 0.06; *Z*=2.42; *P*=.03; psychosomatic problem: from mean 0.40, SEM 0.03 to mean 0.32, SEM 0.03; *Z*=2.66; *P*=.02; impulsivity-hyperactivity: from mean 0.94, SEM 0.06 to mean 0.80, SEM 0.06; *Z*=2.49; *P*=.03; hyperactivity index: from mean 1.06, SEM 0.05 to mean 0.92, SEM 0.05; *Z*=2.90; *P*=.01; n=97). No device-related adverse event or severe adverse event was observed or reported during or after the intervention.

**Conclusions:**

This study preliminarily suggested the significant improvements of ADHD symptoms and attention function after 4 weeks of ADHD-DTx digital therapy combining basic behavioral parent training with satisfying safety outcomes.

## Introduction

Attention-deficit/hyperactivity disorder (ADHD) is a neurodevelopmental disorder that commonly occurs in childhood and is characterized by inattention, hyperactivity, and impulsivity. The primary treatment for ADHD is pharmacotherapy, using methylphenidate, dexamphetamine, or atomoxetine as the first-line drugs [1]. However, pharmacotherapy for ADHD could cause adverse drug reactions such as digestive system issues, irritability, palpitations, and headaches, and more importantly, pharmacotherapy does not target the core functional deficits of patients with ADHD [2]. A recent study also suggested that beginning treatment with behavioral intervention may produce better outcomes overall than beginning treatment with medication [3]. Therefore, many patients with ADHD and their parents are looking forward to alternative interventions [4] like behavior therapy, neurofeedback, counseling, and, more recently, digital therapy [5].

The digital therapy of ADHD has been developed to improve the cognitive functional impairments and attention-control deficits of children with ADHD. In 2020, the US Food and Drug Administration (FDA) approved EndeavorRx (AKL-T01), the first video game–like training software for children with ADHD aged 8-12 years. Growing evidence has suggested that digital therapy could provide a safe and effective intervention to improve functional performance (such as attention, working memory, etc) and problematic behavior of children with ADHD, with minimal risk of adverse events (AEs) compared to pharmacotherapy [6-9]. In 2023, the Chinese National Medical Products Administration (NMPA) approved the “Attention Enhancement Training Software” (ADHD-DTx), a video game–like training software for children with ADHD aged 6-12 years, as the first nationally certified digital therapeutics medical device for ADHD in China.

The neurophysiological mechanisms of EndeavorRx and ADHD-DTx are both based on the self-adaptive multitasking training paradigm (NeuroRacer), which consists of 2 tasks: the “driving task” (sustained attention) and the go/no-go “sign task” (signal detection, attention-shifting, and inhibition control). Multiple studies have revealed that this training paradigm could improve participants’ cognitive control abilities (enhanced sustained attention and working memory) after 1-month training [6-8,10], as well as increase the frontal midline theta (FMϴ) power, the neuromarker of sustained attention and cognitive control [9,10]. Therefore, this paradigm has been used for the intervention of ADHD in a video game–like form to improve compliance in children. Besides the “driving task” and “sign task,” ADHD-DTx also included a third task: the “digit cancellation task” (a widely used attention assessment and training method in clinical practice) [11] to further enhance the training effect of attention function.

To preliminarily evaluate the actual intervention effects of ADHD-DTx in Chinese children with ADHD aged 6-12 years, we performed a single-arm, open-label efficacy and safety study in the Children’s Hospital of Zhejiang University School of Medicine (Hangzhou, China) in 2021. This study provided valuable efficacy and safety data of ADHD-DTx (and ADHD digital therapy based on the self-adaptive multitasking training paradigm) for the first time in Chinese children with ADHD. The efficacy data suggested by this pilot study provided a critical contribution to the design of the following randomized, double-blinded, parallel-controlled clinical trials, which were conducted during 2022 and 2023 (Feng S, PhD, unpublished data, 2025). The Chinese NMPA approval of ADHD-DTx (as the first Chinese ADHD digital therapy medical device) was based on the results of a key Good Clinical Practice clinical trial conducted during 2022-2023 (Feng S, PhD, unpublished data, 2025).

All participants had to be off any ADHD medication and without other significant comorbid psychiatric diagnoses. Included participants were treated with ADHD-DTx therapy and basic behavioral parent training (BPT; positive reinforcement training at home, required according to the ethical consideration of the regulatory agency) [12,13]. Efficacy outcomes included the computerized attention test: Test of Variables of Attention (TOVA) [14,15] and classic scales: (1) the Swanson, Nolan, and Pelham Questionnaire, version 4 (SNAP-IV) [16-18]; (2) the Weiss Functional Impairment Rating Scale (WFIRS) [19]; and (3) the Conner’s Parent Symptom Questionnaire (PSQ) [20,21]. Safety outcomes included the proportions of device-related AEs or severe AEs.

## Methods

### Overview

This study was a single-arm, open-label study in children (aged 6-12 years) with a confirmed diagnosis of ADHD (as per the DSM-5 [*Diagnostic and Statistical Manual of Mental Disorders* {Fifth Edition}) in the Children’s Hospital of Zhejiang University School of Medicine (Hangzhou, China) in 2021. Participants had to be off any ADHD medication and not present other significant comorbid psychiatric diagnoses. All participants had an IQ score of ≥80 (per the Wechsler Intelligence Test) and an Attention-Deficit/Hyperactivity Disorder Rating Scale IV (ADHD-RS-IV) total score of >28.

### Study Design

The study planned to enroll about 100 participants with a confirmed diagnosis of ADHD. Participants received digital therapy (ADHD-DTx, a video game–like training software running on an Android tablet) and basic BPT (positive reinforcement training at home) for 4 weeks (25 min/day, ≥5 times/week). Efficacy outcomes were measured on day 0 (baseline visit) and day 28 (after 4-week treatment). Safety-related events were monitored during and after the trial.

### Participants

Eligible patients were male or female children aged 6-12 years with a confirmed diagnosis of ADHD (as per the DSM-V). Participants had to be off any ADHD medication (for at least 4 weeks before the baseline visit) and not present other significant comorbid psychiatric diagnoses. All participants had an IQ score of ≥80 (per the Wechsler Intelligence Test) and an ADHD-RS-IV total score of ≥28. Complete inclusion and exclusion criteria are in Multimedia Appendix 1.

### Procedures

The ADHD-DTx intervention was preinstalled on Huawei MatePad tablets (Huawei). At the baseline visit, eligible patients were instructed to use ADHD-DTx for about 10 minutes while a study coordinator monitored the session to ensure that patients could follow the rules of ADHD-DTx. Patients were further assessed by ADHD-related scales (symptoms and impairments) and TOVA (attention functioning) at the baseline visit.

Afterward, patients received the ADHD-DTx digital treatment and basic BPT at home for 4 weeks (25 min/day, ≥5 times/week). Basic BPT was required according to the ethical considerations of the regulatory agency. The daily training task consisted of five 4- to 5-minute multitasking missions (total time on task was about 25 minutes). Compliance was monitored remotely using the network by investigators, and daily training reminders were sent to patients’ caregivers manually or automatically. In accordance with a strict, predefined protocol, any participant who failed to complete the required training (25 min/day, ≥5 times/week) and persistently ignored reminders was classified as noncompliant and withdrawn from the trial.

ADHD-DTx is a digital therapeutic that uses a proprietary algorithm designed to improve attention control by training interference management (multitasking). ADHD-DTx mechanisms have been described previously. In brief, users multitask by responding to a perceptual discrimination targeting task and a simultaneous sensory motor navigation task. Users advance by reducing interference costs (closing the performance gap between multitasking and single-tasking), and real-time and periodic recalibration occurs to maintain an optimal difficulty level.

BPT consisted of parent-training courses (focusing on teaching parents specialized child management techniques primarily involving contingency management, such as behavior management principles, parental attending skills and home token system), aerobic exercises (such as jogging, swimming, or rope-skipping 40 min/day, ≥4 times/week), listening-retelling training (the child was asked to accurately retell sentences presented orally by parent, and the sentences were gradually made longer and more detailed to train the child’s auditory attention and memory span), and reading-aloud training (the child was asked to read texts aloud to maintain focus and reduce mind-wandering, thereby promoting the child’s sustained attention) [22-24]. BPT is a regular basic treatment for all children with ADHD in clinical practice.

Safety-related events such as vomiting, dizziness, headache, palpitation, addiction, frustration, eye discomfort, and other similar symptoms were monitored during and after the trial, as described: (1) during the 4-week intervention period, all participants’ parents were contacted daily by a research assistant using online social software to collect safety-related events; and (2) after the intervention period, all participants’ parents were contacted monthly by a research assistant using the telephone for 3 months to collect long-term safety-related events.

### Outcomes

To effectively evaluate the impact of treatment on the core symptoms, problematic behaviors, and functional deficits of children with ADHD, and also ensure the reliability, validity, and objectivity of data, we chose both the computerized attention test and classic scales as the efficacy outcomes.

The primary end point was the improvement of attentional functioning as measured by TOVA from baseline to day 28. TOVA is a computerized, objective test of attentional functioning and has been globally used in clinical and academic institutions [22-24]. The outcomes of TOVA could objectively reflect the functional training effect of ADHD-DTx and provide essential information about the efficacy of the intervention.

Secondary end points included the improvements of classic scales that assess ADHD-related symptoms, functional impairments, and problematic behaviors from baseline to day 28. The SNAP-IV is widely used as the key assessment of ADHD core symptoms, including 3 subsets: (1) inattention (AD), (2) hyperactivity/impulsivity (HD), and (3) oppositional defiant disorder (ODD) [16,17]. WFIRS is a multidimensional, ADHD-specific, functional impairment assessment scale, including 6 domains: family, school, life skills, child’s self-concept, social activities, and risky activities [19]. The PSQ has been widely used to assess problematic behaviors related to ADHD, including 6 domains: conduct problem, learning problem, psychosomatic problem, impulsivity-hyperactivity, anxiety, and hyperactivity index [20,21]. The selection of classic, widely validated scales could provide rich information about the behavioral symptoms, and importantly, the outcomes of these scales could reflect the influence of daily life by the training of ADHD-DTx, which is crucial for the rehabilitation of children with ADHD. More detailed descriptions of the outcome measurements can be found in Multimedia Appendix 2.

### Statistical Analysis

All analyses were performed according to a prespecified statistical analysis plan. Unless otherwise indicated, statistical comparisons used a 2-tailed significance test evaluated at the 95% level of confidence. All analyses were conducted using a complete case analysis. In no situation were missing data to be imputed. Student *t* test was performed only if the analyzed data passed the normality test (Shapiro-Wilk test); otherwise, the Wilcoxon nonparametric test would be used. The chi-square test was used for the statistical inference for counting data. Multiple comparisons corrections (using the false discovery rate method) were performed for all the secondary efficacy end points [25].

The primary efficacy end point for each participant was the change in the TOVA Attention Performance Index (API) from baseline to day 28, defined as the score on day 28 minus the score at baseline. Missing data were not imputed. Participants with missing data either on day 0 or day 28 would be excluded from the paired significance analysis. Unless otherwise indicated, the results of the efficacy analysis were summarized as mean (SE of the mean [SEM]). Significance was assessed with a 2-sided paired *t* test evaluated at the 95% level of confidence.

The following secondary efficacy end points were tested using the same technique outlined for the primary efficacy analysis: (1) change in SNAP-IV from baseline to day 28, (2) change in WFIRS from baseline to day 28, and (3) change in PSQ from baseline to day 28.

### Ethical Considerations

The study was conducted in accordance with the International Conference on Harmonisation Regulations and was approved by the Institutional Review Board of Children’s Hospital of Zhejiang University School of Medicine. All participants and their caregivers provided written informed consent prior to any study activities being conducted. The data of all participants were anonymous throughout the study. All participants received compensation for the examination fees. The study was subject to independent supervision by regulatory agencies (the Institutional Review Board and the Office of Clinical Trial Institution of Children’s Hospital of Zhejiang University School of Medicine) throughout the entire process. Authors who were employed by SDO Digital Therapeutics (the developer of ADHD-DTx) did not participate in any data collection process, and the data analysis results were checked and confirmed by authors from the Children’s Hospital of Zhejiang University School of Medicine. To safeguard the design details and clinical parameters of the investigational product (ADHD-DTx) and mitigate potential commercial risks, the producer requested a delay in the clinical trial registration until after market approval was obtained from the NMPA of China. Following the product’s market approval in 2023, the trial was submitted for registration with the Chinese Clinical Trial Registry, where it is currently pending review.

## Results

### Participants

**Figure 1:**
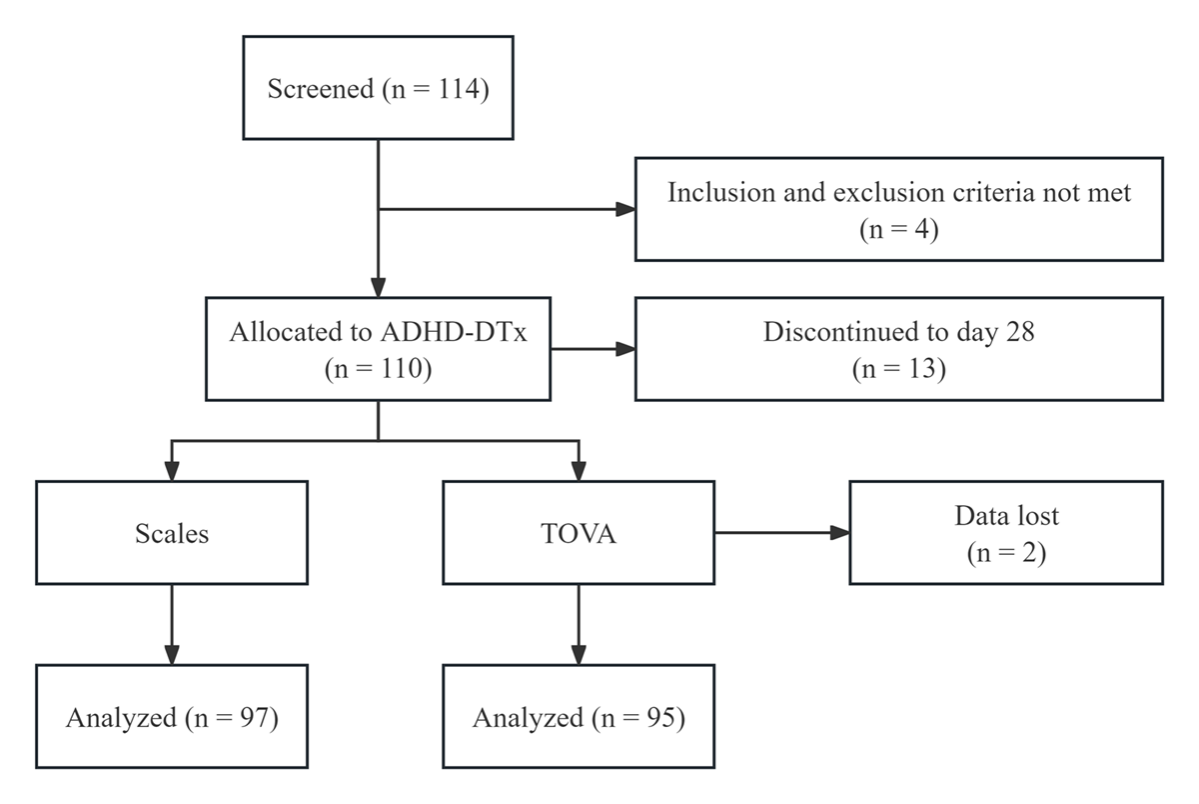
CONSORT (Consolidated Standards of Reporting Trials) flow diagram of the single-arm, open-label study of children with attention-deficit/hyperactivity disorder in China during 2021. TOVA: Test of Variables of Attention.

A total of 114 participants were screened for inclusion in this study, with 110 meeting eligibility criteria (Figure 1). The sample size was determined comprehensively, referring to previous research using similar digital therapy for children with ADHD [8], the power of statistics, and the amount of available resources. The mean age of included participants was 7.78 (SD 1.14) years, and 90% (99/110) were male. The vast majority (106/110, 96.36%) of included participants were of the Han ethnic group, and other involved ethnic groups included She (2/110, 1.82%), Korean (1/110, 0.91%), and Tujia (1/110, 0.91%). The educational status of participants’ parents: postgraduate (6/110, 5.45%), graduate or junior college (62/110, 56.36%), high school or vocational school (28/110, 25.45%), middle school (12/110, 10.91%), primary school, and below (2/110, 1.82%). Demographic characteristics of included participants are listed in Table 1.

**Table 1:** Demographic characteristics of included participants.

Demographic		Value (n=110)	
Age (years), mean (SD)		7.78 (1.14)	
**Gender, n (%)**			
	Male	99 (90)	
	Female	11 (10)	
**Ethnicity, n (%)**			
	Han	106 (96.36)	
	She	2 (1.82)	
	Korean	1 (0.91)	
	Tujia	1 (0.91)	
**Parental education level, n (%)**			
	Postgraduate	6 (5.45)	
	Graduate or junior college	62 (56.36)	
	High school or vocational school	28 (25.45)	
	Middle school	12 (10.91)	
	Primary school and below	2 (1.82)	

Overall, 97/110 (88%) participants completed the study, 13 participants did not complete the study (lost to follow-up or noncompliance), and the TOVA data of 2 participants were lost due to device failure. All 97 participants who completed the study adhered strictly to the training protocol (25 min/day, ≥5 times/week for 4 weeks), and the average number of actual training days completed per participant was 20. Therefore, we finally collected and analyzed 97 participants’ scales data, as well as 95 participants’ TOVA data. All participants received ADHD-DTx digital therapy (combined with basic BPT) in 2021 and remained off medication during the treatment period.

### Efficacy Outcomes

In this study, efficacy outcomes were measured using the computerized attention test (TOVA) and classic scales: SNAP-IV, WFIRS, and PSQ. After a 4-week intervention, we found that the treatment significantly improved ADHD-related symptoms and impairments (all detailed statistical results are present in Table 2).

**Table 2:** Summary of efficacy outcomes during the 4-week treatment.

Efficacy outcome		Participants, n	Day 0, mean (SEM)	Day 28, mean (SEM)	*P* value	Test statistic
**TOVA^a^-API^b^**		95	–4.15 (0.32)	–1.70 (0.30)	<.001	–8.78 (94)^c^
	Younger group	69	–4.15 (0.35)	–1.59 (0.35)	<.001	–7.90 (68)^c^
	Older group	26	–4.15 (0.73)	–2.00 (0.59)	.001	–3.90 (25)^c^
	Male group	84	–4.18 (0.34)	–1.74 (0.31)	<.001	–8.11 (83)^c^
	Female group	11	–3.89 (1.08)	–1.38 (1.19)	.008	–3.31 (10)^c^
**SNAP-IV^d^ total**		97	1.33 (0.05)	1.09 (0.05)	<.001	5.32 (96)^c^
	Younger group	70	1.30 (0.05)	1.09 (0.06)	<.001	4.44 (69)^c^
	Older group	27	1.39 (0.11)	1.11 (0.09)	.007	2.91 (26)^c^
	Male group	86	1.35 (0.05)	1.12 (0.05)	<.001	4.78 (85)^c^
	Female group	11	1.14 (0.08)	0.87 (0.16)	.03	2.59 (10)^c^
**SNAP-IV AD^e^**		97	1.71 (0.06)	1.44 (0.06)	<.001	4.44 (96)^c^
	Younger group	70	1.72 (0.07)	1.42 (0.07)	<.001	4.77 (69)^c^
	Older group	27	1.68 (0.11)	1.51 (0.12)	.23	1.22 (26)^c^
	Male group	86	1.73 (0.06)	1.48 (0.07)	<.001	3.97 (85)^c^
	Female group	11	1.57 (0.11)	1.20 (0.19)	.07	2.04 (10)^c^
**SNAP-IV HD^f^**		97	1.38 (0.07)	1.05 (0.06)	<.001	5.96 (96)^c^
	Younger group	70	1.34 (0.07)	1.07 (0.08)	<.001	4.53 (69)^c^
	Older group	27	1.49 (0.14)	1.01 (0.09)	<.001	3.98 (26)^c^
	Male group	86	1.42 (0.07)	1.07 (0.06)	<.001	5.69 (85)^c^
	Female group	11	1.09 (0.19)	0.89 (0.25)	.09	1.91 (10)^c^
**SNAP-IV ODD^g^**		97	0.84 (0.05)	0.75 (0.05)	.03	2.47^h^
	Younger group	70	0.80 (0.06)	0.74 (0.06)	.17	1.38 (69)^c^
	Older group	27	0.95 (0.13)	0.76 (0.11)	.01	2.50 (26)^c^
	Male group	86	0.86 (0.06)	0.78 (0.06)	.06	1.88 (85)^c^
	Female group	11	0.70 (0.10)	0.47 (0.08)	.04	2.02 (10)^c^
**WFIRS^i^**						
	Family	97	0.75 (0.05)	0.65 (0.04)	.01	2.80^h^
	School	97	0.78 (0.05)	0.72 (0.04)	.14	1.55^h^
	Life skills	97	0.72 (0.04)	0.65 (0.04)	.09	1.85^h^
	Child’s self-concept	97	0.65 (0.06)	0.59 (0.05)	.34	0.96^h^
	Social activities	97	0.56 (0.05)	0.45 (0.05)	.01	2.91^h^
	Risky activities	97	0.27 (0.03)	0.23 (0.03)	.14	1.54^h^
**PSQ^j^**						
	Conduct problem	97	0.82 (0.05)	0.74 (0.05)	.06	2.04^h^
	Learning problem	97	1.72 (0.06)	1.57 (0.06)	.03	2.42^h^
	Psychosomatic problem	97	0.40 (0.03)	0.32 (0.03)	.02	2.66^h^
	Anxiety	97	0.36 (0.04)	0.34 (0.04)	.17	1.40^h^
	Impulsivity-hyperactivity	97	0.94 (0.06)	0.80 (0.06)	.03	2.49^h^
	Hyperactivity index	97	1.06 (0.05)	0.92 (0.05)	.01	2.90^h^

^a^TOVA: Test of Variables of Attention.

^b^API: Attention Performance Index.

^c^*t* test (*df*).

^d^SNAP-IV: Swanson, Nolan, and Pelham Questionnaire, version 4.

^e^AD: inattention subscale of SNAP-IV.

^f^HD: hyperactivity/impulsivity subscale of SNAP-IV.

^g^ODD: oppositional defiant disorder subscale of SNAP-IV.

^h^*Z* value of Wilcoxon nonparametric test.

^i^WFIRS: Weiss Functional Impairment Rating Scale.

^j^PSQ: Conner’s Parent Symptom Questionnaire.

#### Primary End Point: TOVA-API

**Figure 2:**
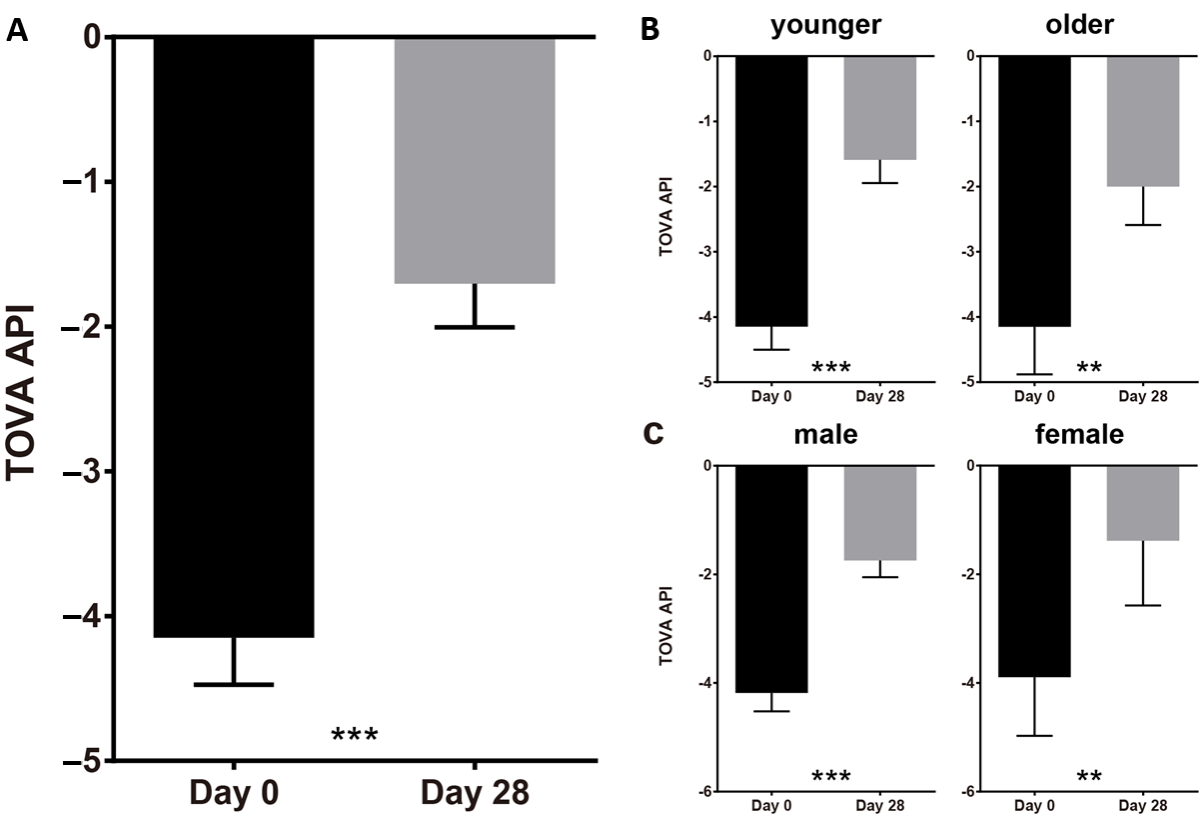
Statistical results of the Test of Variables of Attention (TOVA) suggested significant improvement after 4 weeks of intervention. (A) TOVA Attention Performance Index (API) outcomes from day 0 to day 28; (B) TOVA-API of younger (aged 6-8 years) and older (aged 9-12 years) groups from day 0 to day 28; (C) TOVA-API of male and female groups from day 0 to day 28. Error bars indicate SEM. **P<.01, ***P<.001.

We chose TOVA (an objective measurement of attention function) as the primary measurement of intervention efficacy, and the TOVA-API was chosen as the primary end point of this study (Figure 2).

The baseline data of TOVA-API passed the normality test (n=95; *P*=.36). From day 0 (baseline) to day 28, the population TOVA-API exhibited statistically significant improvement (Figure 2A, from mean –4.15, SEM 0.32 to mean –1.70, SEM 0.30; T=–8.78; n=95; *P*<.001), suggesting the efficacy of objective functional improvement of attention.

We conducted subgroup analysis of TOVA-API on age (Figure 2B; younger: 6-8 years; older: 9-12 years) and gender (Figure 2C; male and female) to investigate more detailed efficacy characteristics. We found that from day 0 (baseline) to day 28, both the younger and older groups exhibited statistically significant improvement (younger group: from mean –4.15, SEM 0.35 to mean –1.59, SEM 0.35; T=–7.90; n=69; *P*<.001; older group: from mean –4.15, SEM 0.73 to mean –2.00, SEM 0.59; T=–3.90; n=26; *P*=.001). The improvement of the younger group (pre-post difference=2.56) slightly exceeded that of the older group (pre-post difference=2.15), but no statistical difference was found (*P*=.53). As for the gender analysis, from day 0 (baseline) to day 28, both the male and female groups exhibited statistically significant improvement (male group: from mean –4.18, SEM 0.34 to mean –1.74, SEM 0.31; T=–8.11; n=84; *P*<.001; female group: from mean –3.89, SEM 1.08 to mean –1.38, SEM 1.19; T=–3.31; n=11; *P*=.008). The improvements of both groups showed no statistical difference (*P*=.93). We also analyzed the potential influence of parental education level (college degree or above: n=56; below college level: n=39) on TOVA-API and found no statistically significant difference (*P*=.78).

The above findings suggested that the 4-week intervention could significantly improve the attention function of children with ADHD (regardless of age or gender), as measured by TOVA-API.

#### Secondary End Points: SNAP-IV, WFIRS, and PSQ

We chose the results of classic clinical scales (SNAP-IV, WFIRS, and PSQ) as the secondary end points. Multiple comparisons corrections were performed for the statistical analysis results of the scale scores (see Methods section).

**Figure 3:**
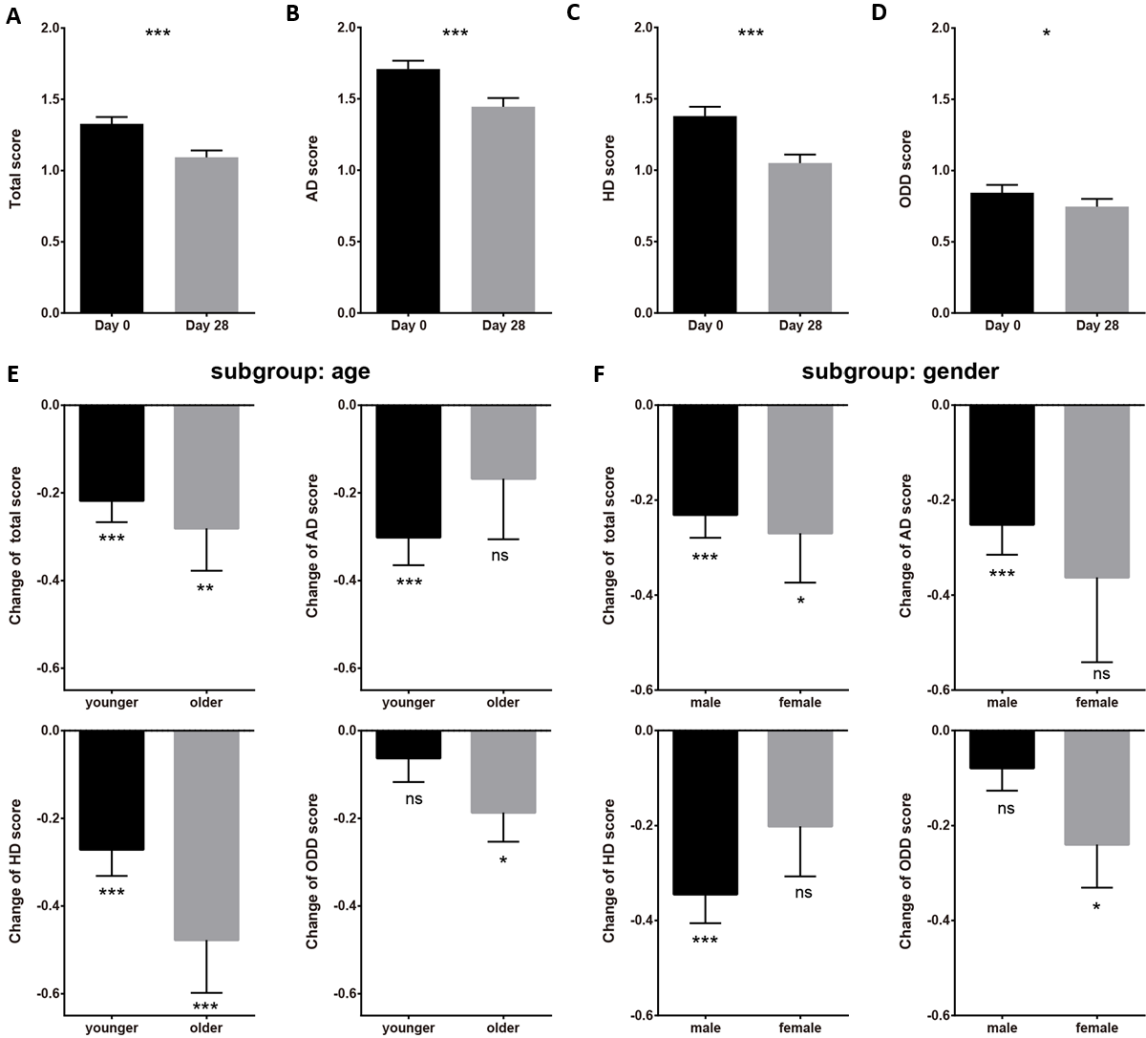
Statistical results of the Swanson, Nolan, and Pelham Questionnaire, version 4 (SNAP-IV), suggested significant improvement after 4 weeks of intervention. (A) SNAP-IV total score from day 0 to day 28; (B) SNAP-IV inattention (AD) score from day 0 to day 28; (C) SNAP-IV hyperactivity/impulsivity (HD) score from day 0 to day 28. (D) SNAP-IV oppositional defiant disorder (ODD) score from day 0 to day 28. (E) Subgroup analysis of age (younger: 6-8 years; older: 9-12 years) of SNAP-IV scores from day 0 to day 28. (F) Subgroup analysis of gender (male and female) of SNAP-IV scores from day 0 to day 28. Error bars indicate SEM. ns: nonsignificant. *P<.05, **P<.01, ***P<.001.

Using the SNAP-IV scale, we measured the treatment effect of ADHD core symptoms (Figure 3). The baseline data of the SNAP-IV total score, AD subscale, and HD subscale passed the normality test (n=97; *P*_total_=.19; *P*_AD_=.09; *P*_HD_=.25), while that of the ODD subscale did not (n=97; *P*_ODD_<.001). Therefore, we used the Student *t* test for total score, AD, and HD data, and the Wilcoxon nonparametric test for ODD data.

From day 0 to day 28, the population total score of SNAP-IV significantly improved (descended) from mean 1.33, SEM 0.05 to mean 1.09, SEM 0.05 (Figure 3A; T=5.32; n=97; *P*<.001); the population AD score of SNAP-IV significantly improved (descended) from mean 1.71, SEM 0.06 to mean 1.44, SEM 0.06 (Figure 3B; T=4.44; n=97; *P*<.001); the population HD score of SNAP-IV significantly improved (descended) from mean 1.38, SEM 0.07 to mean 1.05, SEM 0.06 (Figure 3C; T=5.96; n=97; *P*<.001); the population ODD score of SNAP-IV significantly improved (descended) from mean 0.84, SEM 0.05 to mean 0.75, SEM 0.05 (Figure 3D; *Z*=2.47; n=97; *P*=.03). The above results suggested that the treatment could significantly improve the core symptoms (attention deficit and hyperactivity) of children with ADHD.

We also conducted subgroup analysis of SNAP-IV on age (Figure 3E; younger: 6-8 years; older: 9-12 years) and gender (Figure 3F; male and female). It was notable that due to the small sample size of the female group (n=11) and older group (n=27), the efficacy of statistical inference might be limited.

For the SNAP-IV total score, all the subgroups exhibited statistically significant improvement (younger group: from mean 1.30, SEM 0.05 to mean 1.09, SEM 0.06; T=4.44; n=70; *P*<.001; older group: from mean 1.39, SEM 0.11 to mean 1.11, SEM 0.09; T=2.91; n=27; *P*=.007; male group: from mean 1.35, SEM 0.05 to mean 1.12, SEM 0.05; T=4.78; n=86; *P*<.001; female group: from mean 1.14, SEM 0.08 to mean 0.87, SEM 0.16; T=2.59; n=11; *P*=.03 paired *t* test). No statistical difference was found between different age and gender groups (age: *P*=.56; gender: *P*=.74).

For the SNAP-IV AD score, only the younger group and male group exhibited statistically significant improvement (younger group: from mean 1.72, SEM 0.07 to mean 1.42, SEM 0.07; T=4.77; n=70; *P*<.001; male group: from mean 1.73, SEM 0.06 to mean 1.48, SEM 0.07; T=3.97; n=86; *P*<.001), while the older group and female group did not (older group: from mean 1.68, SEM 0.11 to mean 1.51, SEM 0.12; T=1.22; n=27; *P*=.23; female group: from mean 1.57, SEM 0.11 to mean 1.20, SEM 0.19; T=2.04; n=11; *P*=.07). No statistical difference was found between different age and gender groups (age: *P*=.39; gender: *P*=.57).

For the SNAP-IV HD score, the younger, older, and male groups exhibited statistically significant improvement (younger group: from mean 1.34, SEM 0.07 to mean 1.07, SEM 0.08; T=4.53; n=70; *P*<.001; older group: from mean 1.49, SEM 0.14 to mean 1.01, SEM 0.09; T=3.98; n=27; *P*<.001; male group: from mean 1.42, SEM 0.07 to mean 1.07, SEM 0.06; T=5.69; n=86; *P*<.001), while the female group did not (n=11; *P*=.09). No statistical difference was found between different age and gender groups (age: *P*=.94; gender: *P*=.26).

For the SNAP-IV ODD score, only the older group and female group exhibited statistically significant improvement (older group: from mean 0.95, SEM 0.13 to mean 0.76, SEM 0.11; T=2.50; n=27; *P*=.01; female group: from mean 0.70, SEM 0.10 to mean 0.47, SEM 0.08; T=2.02; n=11; *P*=.04), while the younger group and male group did not (younger group: T=1.38; n=70; *P*=.17; male group: T=1.88; n=86; *P*=.06). No statistical difference was found between different age and gender groups (age: *P*=.17; gender: *P*=.22).

**Figure 4:**
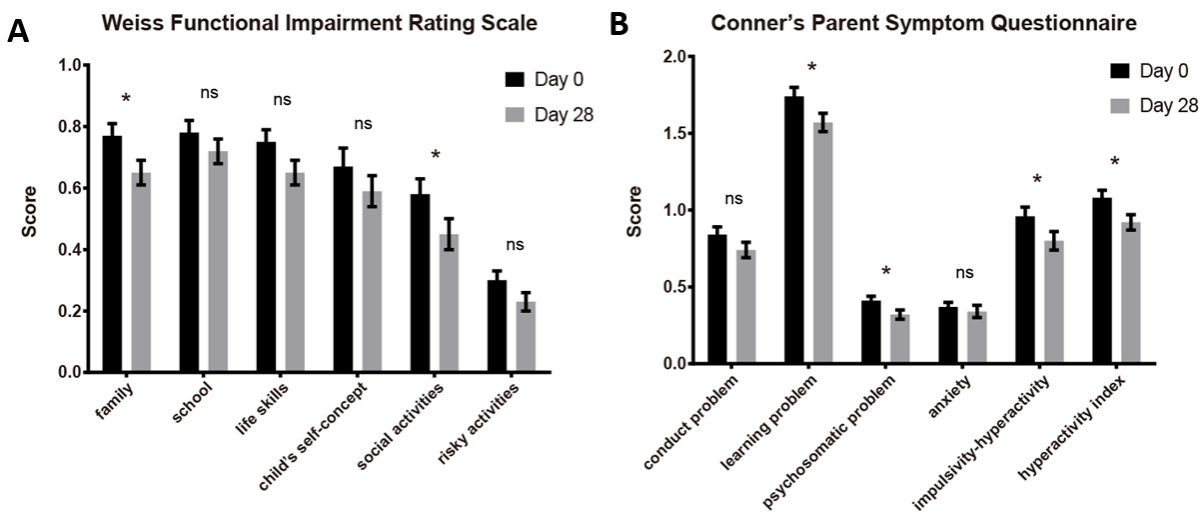
Statistical results of the Weiss Functional Impairment Rating Scale and Conner’s Parent Symptom Questionnaire suggested significant improvement after 4 weeks of intervention. (A) Weiss Functional Impairment Rating Scale outcomes (scores) from day 0 to day 28. (B) Conner’s Parent Symptom Questionnaire outcomes (scores) from day 0 to day 28. Error bars indicate SEM. ns: nonsignificant. *P<.05, **P<.01, ***P<.001.

The functional impairments of participants were measured using WFIRS (Figure 4A). WFIRS data did not pass the normality test; therefore, the Wilcoxon signed-rank test was used. We found that after 4 weeks of treatment, the impairments of the “family” and “social activities” domains were statistically significantly improved (family: from mean 0.75, SEM 0.05 to mean 0.65, SEM 0.04; *Z*=2.80; *P*=.01; social activities: from mean 0.56, SEM 0.05 to mean 0.45, SEM 0.05; *Z*=2.91; n=97; *P*=.01), while the domains of “school,” “life skills,” “child’s self-concept,” and “risky activities” exhibited nonsignificant improvement (school: *P*=.14; life skills: *P*=.09; child’s self-concept: *P*=.34; risky activities: *P*=.14; n=97).

We also analyzed the number of items scored ≥2 in WFIRS to investigate the treatment effect on severe functional impairments using the chi-square test. We found that the correlation between the intervention and the number of items scored ≥2 of “child’s self-concept” was statistically significant (Pearson *χ*^2^_1_=7.249; *P*=.007). There were also almost significant correlations between the intervention and the number of items scored ≥2 of “family” and “life skills” (family: Pearson *χ*^2^_1_=3.183; *P*=.07; life skills: Pearson *χ*^2^_1_=3.071; *P*=.08). While there were no significant correlations between the intervention and the number of items scored ≥2 of “school,” “social activities,” and “risky activities” (school: Pearson *χ*^2^_1_=0.949; *P*=.33; social activities: Pearson *χ*^2^_1_=0.899; *P*=.34; risky activities: Pearson *χ*^2^_1_=0.519; *P*=.47).

The problematic behaviors of participants were measured using PSQ (Figure 4B). PSQ data did not pass the normality test; therefore, the Wilcoxon signed-rank test was used. After 4 weeks treatment, the domains of “learning problem,” “psychosomatic problem,” “impulsivity-hyperactivity,” and “hyperactivity index” were statistically significantly improved (learning problem: from mean 1.72, SEM 0.06 to mean 1.57, SEM 0.06; *Z*=2.42; *P*=.03; psychosomatic problem: from mean 0.40, SEM 0.03 to mean 0.32, SEM 0.03; *Z*=2.66; *P*=.02; impulsivity-hyperactivity: from mean 0.94, SEM 0.06 to mean 0.80, SEM 0.06; *Z*=2.49; *P*=.03; hyperactivity index: from mean 1.06, SEM 0.05 to mean 0.92, SEM 0.05; *Z*=2.90; *P*=.01; n=97). While the domains of “conduct problem” and “anxiety” exhibited nonsignificant improvement (conduct problem: *P*=.06; anxiety: *P*=.17).

The above findings suggested that the 4-week intervention could significantly improve the core symptoms of children with ADHD (measured by SNAP-IV), as well as a few domains related to functional impairments and problematic behaviors (measured by WFIRS and PSQ).

**Figure 5:**
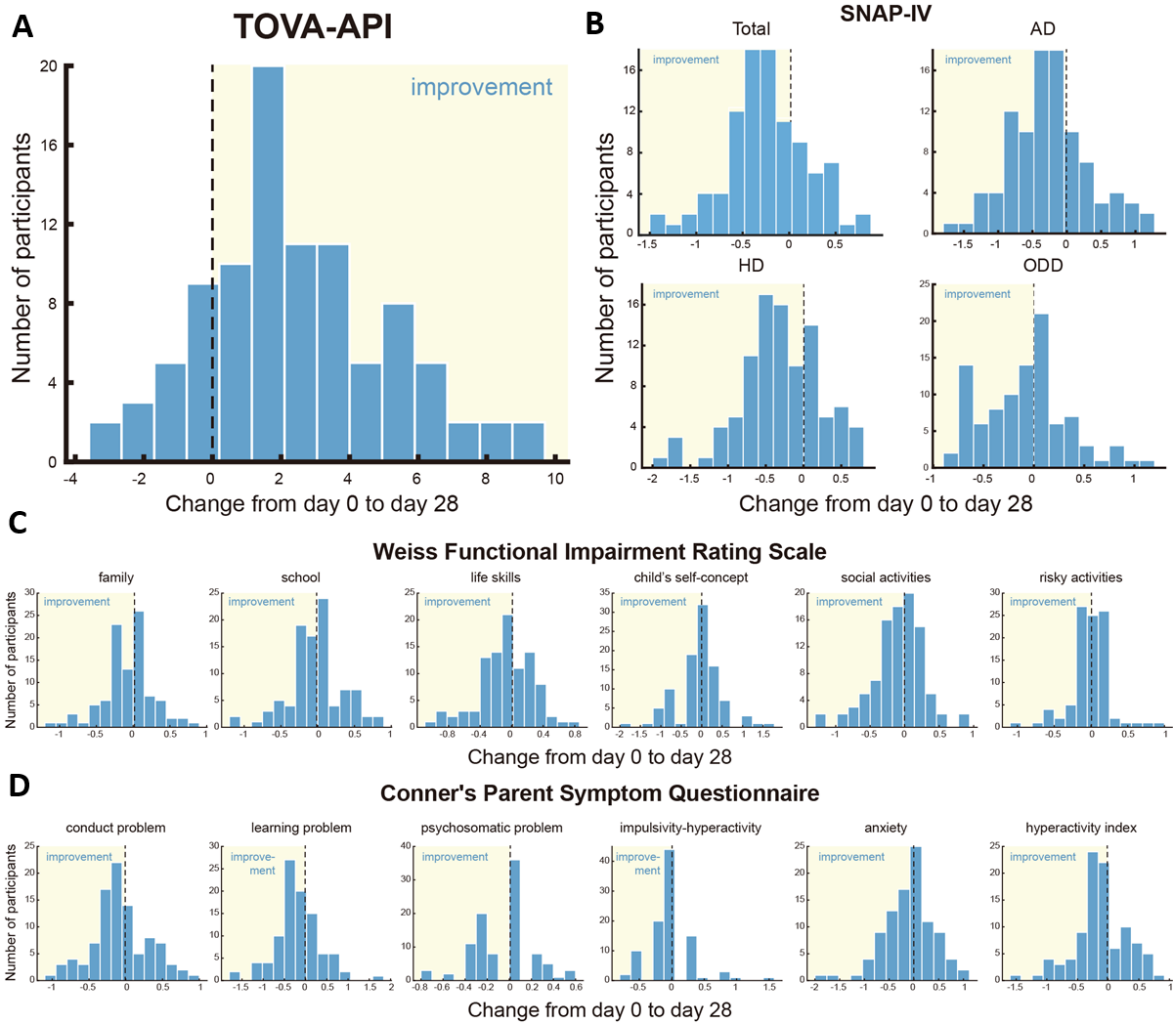
Distributions of efficacy-outcome improvements (from day 0 to day 28) among the participants suggest significant improvement of symptoms. (A) Distribution of Test of Variables of Attention (TOVA) Attention Performance Index (API) changes from day 0 to day 28; (B) Distributions of Swanson, Nolan, and Pelham Questionnaire, version 4 (SNAP-IV) score changes from day 0 to day 28; (C) Distributions of Weiss Functional Impairment Rating Scale score changes from day 0 to day 28; (D) Distributions of Conner’s Parent Symptom Questionnaire score changes from day 0 to day 28. The ranges corresponding to improvement are highlighted. AD: inattention; HD: hyperactivity/impulsivity; ODD: oppositional defiant disorder.

Table 2 summarizes the statistical results of efficacy outcomes during the 4-week treatment. The distributions of efficacy-outcome improvements (from day 0 to day 28) among the participants are shown in Figure 5. The proportions of participants whose efficacy outcomes showed improvements were 80% (76/95 in TOVA), 71.13% (69/97 in SNAP-IV total), 70.1% (68/97 in SNAP-IV AD), 70.1% (68/97 in SNAP-IV HD), 55.67% (54/97 in SNAP-IV ODD), 54.64% (53/97 in WFIRS-family), 52.58% (51/97 in WFIRS-school), 52.58% (51/97 in WFIRS-life skills), 38.14% (37/97 in WFIRS-child’s self-concept), 54.64% (53/97 in WFIRS-social activities), 42.27% (41/97 in WFIRS-risky activities), 58.76% (57/97 in PSQ-conduct problem), 48.45% (47/97 in PSQ-learning problem), 45.36% (44/97 in PSQ-psychosomatic problem), 32.99% (32/97 in PSQ-anxiety), 47.42% (46/97 in PSQ-impulsivity-hyperactivity), and 57.73% (56/97 in PSQ-hyperactivity index).

### Safety Results

Parents and participants were informed to report any safety concerns and discomfort during the study. Any concerns and discomfort reported by parents and participants during the study were recorded. Overall, no device-related AE or severe AE was observed or reported during the 4-week intervention and the 3-month follow-up period, suggesting the safety of treatment.

## Discussion

### Principal Findings

This paper reports the first Chinese clinical trial outcomes of an adaptive multitasking training paradigm for the treatment of children with ADHD without medication. This study preliminarily suggested the significant improvements of ADHD symptoms and attention function after 1-month digital therapy (combined with basic BPT, without medication) using the “Attention Enhancement Training Software” (ADHD-DTx), a video game–like training software for children with ADHD aged 6-12 years, with satisfying safety outcomes.

### Comparison to Prior Work

The efficacy results are consistent with previous studies using an adaptive multitasking training paradigm [7,8,10], indicating the potential application value (as an individual or adjuvant treatment) of video game–like digital therapy for children with ADHD. The potential contribution of game-like cognitive training on attention has been reported both in children and adults [26-30], which suggests a new digital solution for attention-related deficits (such as ADHD, mild cognitive impairment, etc) across age groups [31,32].

The BPT has been extensively studied, and the overall effect size was estimated by previous researchers. Gubbels and colleagues [33] suggested that the overall effect of parent training was 0.416 (Cohen *d*) in their meta-analysis research, while Zwi and colleagues [23] suggested that the effect size might be “between small and medium” in their review. In comparison, we calculated the effect size (Cohen *d*) of the intervention in this study, based on TOVA-API and SNAP-IV data. We found that the effect size of TOVA-API was 0.80, while that of SNAP-IV total score was 0.50. These results exceeded the effect sizes of BPT estimated by previous researchers, suggesting that the ADHD-DTx digital therapeutic, when integrated with BPT, may yield superior outcomes to BPT alone. However, such cross-study comparisons are informal and cannot independently establish the efficacy of ADHD-DTx. More rigorously controlled trials—directly comparing the combined intervention against BPT alone—are needed to draw definitive conclusions regarding the stand-alone contribution of the digital therapy.

Compared with the FDA-approved EndeavorRx (AKL-T01) software, the “Attention Enhancement Training Software” (ADHD-DTx) added the “digit cancellation task” (a widely used attention assessment and training method in clinical practice [11]) to further enhance the training effect on attention function. The “digit cancellation task” was simplified from the clinical version and consisted of 5 different types of tasks: (1) choose a specific number (eg, “choose 3”), (2) choose the adjacent (left or right) number of a specific number (eg, “choose the adjacent number on the left of 3”), (3) choose a specific number adjacent (left or right) to a specific number (eg, “choose 2 on the left of 3”), (4) choose the number in the middle position of 2 specific numbers (eg, “choose the number in the middle position of 2 and 3”), and (5) choose a specific type (even or odd) of number in the middle position of 2 specific numbers (eg, “choose the even number in the middle position of 2 and 3”). These tasks targeted multiple dimensions of attention (such as pointing, shifting, selection, span, and allocation), aiming to activate extensive attention-related brain regions and achieve the training effect of attention function.

### Limitations

The key objective of this study was to explore the clinical safety and efficacy of ADHD-DTx digital therapy. However, considering that this study was single-armed and did not use a control group, it would be meaningful to attempt to exclude the influence of basic BPT from the final therapeutic effect. We suggest that further research should use a randomized, double-blinded, parallel-controlled design, which could provide the most reliable data reflecting the actual effect of ADHD-DTx.

In this study, several potential confounding factors should be considered. First, the subjective evaluation bias was caused by the open-label design, the subjective parent-reported scales for all secondary outcomes, and the large research time span. Open-label design and subjective scales could introduce a significant risk of reporter bias or placebo effect, while the 1-year time span of this study might further enhance the potential subjective bias due to the fluctuating behavior patterns of children between school and vacation periods. These confounding factors could not be separated from the intervention’s true effect, and future studies should adopt a double-blind, randomized controlled design to mitigate these biases.

Second, heterogeneity in the effectiveness of BPT may have contributed to variability in outcomes. The actual effect of BPT largely depended on the performance of parents when providing behavioral guidance to children with ADHD. However, due to differences in educational level, professional skills, and training quality, the actual effectiveness may vary among different parents, resulting in population heterogeneity. During the study, all parents were asked to daily report their home-based training progress, and all participants exhibited high adherence to both ADHD-DTx and BPT. And the subgroup analysis focusing on parental education level did not show a significant difference in efficacy outcome. Therefore, the potential influence of adherence and parental education level may be limited. However, the potential impact of training quality differences between parents still existed.

Third, the potential contribution of BPT was not excluded in this single-arm study design, which might amplify the effectiveness results. As a standard foundational treatment for ADHD, BPT was mandated by the regulatory agency for all participants due to ethical considerations. While its efficacy was well-established, albeit generally mild, its concurrent implementation with the investigational digital therapy (ADHD-DTx) introduced a considerable confounding effect that may inflate the perceived effectiveness of the intervention. Improved study design should include a control group (providing BPT equally to both test and control groups, while only the test group is treated with ADHD-DTx) so as to effectively control the confounding influence of BPT, allowing for a more precise assessment of the benefit of ADHD-DTx.

Fourth, the generalizability of the results was constrained due to limited sample diversity. The included participants were predominantly male (99/110, 90%) and of Han ethnicity (106/110, 96.36%), which weakened the interpretability of outcomes for females and other ethnicities. Future research should prioritize enrolling more representative samples with balanced gender distribution and greater ethnic diversity to ascertain the efficacy of the intervention across the broader population with ADHD.

Fifth, the rigorously monitored and controlled conditions of a clinical trial may not perfectly predict the intervention’s effectiveness in real-world clinical practice. The high adherence observed in this study was facilitated by intensive monitoring and support, which limited the direct translation of these efficacy results into real-world practice, where effectiveness might be weaker. Consequently, future investigation should involve pragmatic trials to generate robust evidence on the actual efficacy and implementation of the intervention.

Finally, the subgroup analyses for age and gender were likely underpowered due to the small sample sizes in the female and older groups. Consequently, any observed differences (or lack thereof) between these subgroups should be considered preliminary and interpreted with caution. Further research specifically designed to investigate these demographic groups is needed to advance our understanding of digital therapeutics for ADHD.

### Future Directions

Further improvements could still be carried out in future research. In this single-arm, open-label study, we preliminarily revealed the safety and efficacy of ADHD-DTx digital therapy. While future research should use a randomized, double-blinded, parallel controlled design so as to prevent potential placebo effect and rule out the effect of basic BPT at home, it will achieve a better evaluation of the actual effect size of ADHD-DTx. Further study could also investigate the effect of a prolonged treatment period (>4 weeks) to study the long-term influence of digital therapy. Longer safety monitoring after the intervention period is also needed to reveal potential effects on visual acuity, daily activities, sleep quality, screen usage time, and so on. The independent ethical oversight should continue to be valued and implemented to avoid potential conflicts of interest. Future studies should include comparisons with well-established interventions like pharmacotherapy, psychological behavioral therapy, and so on, to obtain more informative evidence for better clinical practice.

There is psychological and neurophysiological evidence suggesting the potential mechanisms of ADHD treatment by the adaptive multitasking paradigm. According to electroencephalogram studies focusing on the neural mechanisms of the adaptive multitasking paradigm, after 4 weeks of training, participants’ FMϴ significantly enhanced [9,10]. The FMϴ is a well-established neural marker of attention control [34-38], and its enhancement is related to the suppression of a key node of the “default mode network” [10,39,40], leading to a reduction of the susceptibility to internal distraction, resulting in better task performance and sustained attention. To explore potential neural mechanisms of digital therapy, physiological measurements of brain function (such as electroencephalogram, functional magnetic resonance imaging, functional near-infrared spectroscopy, functional ultrasound, etc) could provide irreplaceable insight and should be considered in future studies.

The evaluation methods used in this study were limited, and further study could include other powerful tools such as other neuropsychological tests (eg, the Integrated Visual and Auditory Continuous Performance Test, the Wisconsin Card Sorting Test, the Cambridge Neuropsychological Test Automatic Battery, etc), classic scales (eg, the Execution Function Parent Questionnaire, the Vanderbilt ADHD Parent Rating Scale, etc).

Due to the limited data on female patients, we suggest that further research could include a higher proportion of female patients so as to make the results more representative. The inclusion and exclusion criteria set strict restrictions on comorbidity, which, on the one hand, could reduce potential confounding factors; however, on the other hand, it would limit our knowledge of real-world situations. We suggest that subsequent research can specifically investigate the effect of ADHD digital therapy involving comorbidities. We also suggest including the examination of visual acuity in further study to investigate the potential impact of long-term electronic device training on vision.

### Conclusions

According to our pilot study on the Attention Enhancement Training Software (ADHD-DTx), we suggest that it could be used as a daily home-training tool for children with ADHD as an adjunct therapy to medication or other behavioral therapies, thereby further improving the intervention efficacy and reducing potential adverse drug reactions.

## Appendix

Multimedia Appendix 1 Complete inclusion and exclusion criteria.

Multimedia Appendix 2 Description of outcome measurements.

## Abbreviations

AD: inattention subscale of SNAP-IV
ADHD: attention-deficit/hyperactivity disorder
ADHD-RS-IV: Attention-Deficit/Hyperactivity Disorder Rating Scale IV
AE: adverse event
API: Attention Performance Index
BPT: behavioral parent training
DSM-V: Diagnostic and Statistical Manual of Mental Disorders, Fifth Edition
FDA: US Food and Drug Administration
FMϴ: frontal midline theta
HD: hyperactivity/impulsivity subscale of SNAP-IV
NMPA: National Medical Products Administration
ODD: oppositional defiant disorder subscale of SNAP-IV
PSQ: Conner’s Parent Symptom Questionnaire
SEM: SE of the mean
SNAP-IV: Swanson, Nolan, and Pelham Questionnaire, version 4
TOVA: Test of Variables of Attention
WFIRS: Weiss Functional Impairment Rating Scale

## Data Availability

The data that support the findings of this study are available on request from the corresponding author upon reasonable request.
