# Supplementary material for: Efficacy and Safety of a Video Game–Like Digital Therapy Intervention for Chinese Children With Attention-Deficit/Hyperactivity Disorder: Single-Arm, Open-Label Pre-Post Study
Source: JMIR Serious Games. 2026 Jan 5;14:e76114. doi: 10.2196/76114 (PMC12817042; doi:10.2196/76114)
Supplement: Multimedia Appendix 1 [file games_v14i1e76114_app1.docx]

**Complete inclusion and exclusion criteria (Appendix 1)**

**Inclusion criteria**

1. Informed consent from participants and caregivers;
2. Aged 6-12 years (including 12 years);
3. Diagnosis of ADHD (according to DSM-V) by doctors through assessments and interviews;
4. ADHD-RS-IV total score ≥ 28 at the screening/baseline examination;
5. Not using pharmacotherapy for ADHD treatment;
6. The estimated IQ score ≥ 80 (Wechsler Intelligence Test).

**Exclusion criteria**

1. Diagnosis of comorbidity requiring drug control, or uncontrolled severe mental illness, including (but not limited to) post-traumatic stress disorder, bipolar disorder, generalized developmental disorder, severe obsessive-compulsive disorder, severe depression disorder, conduct disorder, etc;
2. Children who are currently at risk of suicide, have attempted suicide, or have a history of suicide;
3. Physical movement conditions that hinder gaming (such as hand/arm deformities, prosthetics, etc);
4. Recent (within the past 6 months) suspected history of drug abuse or dependence;
5. History of epilepsy (excluding febrile seizures), or obvious motor or vocal convulsions, including (but not limited to) Tourette disorder;
6. Children with color blindness;
7. Extremely weak or uncorrected vision;
8. Children with severe intellectual disabilities;
9. Received medication treatment with methylphenidate, amphetamine or tomoxetine within 4 weeks prior to screening;
10. Received DHA related health products or medication treatment within 4 weeks prior to screening;
11. Received traditional Chinese medicines with clear indications for ADHD within 4 weeks prior to screening.
