# Supplementary material for: Efficacy and Safety of a Video Game–Like Digital Therapy Intervention for Chinese Children With Attention-Deficit/Hyperactivity Disorder: Single-Arm, Open-Label Pre-Post Study
Source: JMIR Serious Games. 2026 Jan 5;14:e76114. doi: 10.2196/76114 (PMC12817042; doi:10.2196/76114)
Supplement: Multimedia Appendix 2 [file games_v14i1e76114_app2.docx]

**Description of Outcome Measurements (Appendix 2)**

| **Outcome Measurement** | **Description** |
| --- | --- |
| TOVA | TOVA is a computerized, objective test of attentional functioning, and has been globally used in clinical and academic institutions. |
| - API | The Attention Performance Index (API) is the principal TOVA outcome, representing a composite score of overall attentional functioning used in clinical assessments. |
| SNAP-IV | The Swanson, Nolan, and Pelham Questionnaire, version 4 (SNAP-IV) is widely used as the key assessment of ADHD core-symptoms. |
| - total | The overall assessment of ADHD core-symptoms. |
| - AD | The assessment of inattention symptoms of ADHD. |
| - HD | The assessment of hyperactivity/impulsivity symptoms of ADHD. |
| - ODD | The assessment of opposition/defiance symptoms of ADHD. |
| WFIRS | The Weiss Functional Impairment Rating Scale (WFIRS) is a multi-dimensional, ADHD-specific, functional impairment assessment scale, including 6 domains. |
| - family | The assessment of family-related functional impairments that causes family conflict and difficulties in family interactions. |
| - school | The assessment of school-related functional impairments that causes troubles at school and difficulties in learning. |
| - life skills | The assessment of life-skills-related functional impairments that causes difficulties in daily routine, diet, sleep and physical health. |
| - child’s self-concept | The assessment of self-concept-related functional impairments that causes low self-evaluation and lack of happiness. |
| - social activities | The assessment of social-activities-related functional impairments that causes peer conflict and difficulties in social interactions. |
| - risky activities | The assessment of risky-activities-related functional impairments that causes problematic, dangerous or illegal behaviors. |
| PSQ | The Conner’s Parent Symptom Questionnaire (PSQ) has been widely used to assess problematic behaviors related to ADHD, including 6 domains. |
| - conduct problem | The assessment of problematic behaviors related to conduct, such as rudeness, aggressiveness, opposition/defiance and illegal behaviors. |
| - learning problem | The assessment of problematic behaviors related to learning, such as distraction or giving up during learning. |
| - psychosomatic problem | The assessment of problematic behaviors related to psychosomatic health, such as headache, sleep disorder and stomachache. |
| - anxiety | The assessment of problematic behaviors related to anxiety, such as conflict with unfamiliar environment and excessive worry. |
| - impulsivity-hyperactivity | The assessment of problematic behaviors related to impulsivity/hyperactivity, such as excessive body movements and emotional impulsivity. |
| - hyperactivity index | The comprehensive assessment of hyperactivity that integrates multiple behavioral domains, serves as a frequent tool for preliminary ADHD clinical evaluation. |
